# Association between Habitual Dietary Iron Intake and Glucose Metabolism in Individuals after Acute Pancreatitis

**DOI:** 10.3390/nu12113579

**Published:** 2020-11-22

**Authors:** Wandia Kimita, Xinye Li, Juyeon Ko, Sakina H. Bharmal, David Cameron-Smith, Maxim S. Petrov

**Affiliations:** 1 School of Medicine, University of Auckland, Auckland 1023, New Zealand; wandia.kimita@auckland.ac.nz (W.K.); xli949@aucklanduni.ac.nz (X.L.); ju.ko@auckland.ac.nz (J.K.); s.bharmal@auckland.ac.nz (S.H.B.); 2 Singapore Institute for Clinical Sciences, Agency for Science, Technology and Research, Singapore 117609, Singapore; dcameron_smith@sics.a-star.edu.sg

**Keywords:** dietary iron, haem iron, non-haem iron, acute pancreatitis, haemoglobin A1c, fasting plasma glucose

## Abstract

Dietary intake of iron is known to be associated with impaired glucose metabolism. However, its involvement in derangements of glucose metabolism after acute pancreatitis (AP) is not completely understood. The aim was to investigate the association between dietary iron intake and markers of glucose metabolism in individuals after an attack of AP. Fasting blood samples were collected to analyse markers of glucose metabolism (fasting plasma glucose (FPG) and haemoglobin A1c (HbA1c)). The EPIC-Norfolk food frequency questionnaire was used to determine the habitual intake of dietary iron (total, haem, and non-haem). Multivariable linear regression analyses were conducted and six statistical models were built to adjust for covariates. A total of 109 individuals after AP were studied in a cross-sectional fashion. Total iron (β (95% confidence interval) = −0.19 (−0.35, −0.05); *p* = 0.01 in the most adjusted model) and non-haem iron (β (95% confidence interval) = −0.19 (−0.33, −0.04); *p* = 0.03 in the most adjusted model) were significantly associated with FPG, consistently in all adjusted model. Total iron and non-haem iron did not have consistent significant associations with HbA1c. Dietary haem iron intake was not associated with either FPG or HbA1c. Habitual intake of dietary iron is inversely associated with FPG in individuals after an attack of AP and may be involved in the pathogenesis of new-onset diabetes after pancreatitis. Prospective longitudinal studies are now warranted to unveil the specific mechanism underlying the involvement of dietary iron.

## 1. Introduction

Iron is thought to play a role in the development of a broad spectrum of metabolic derangements (e.g., dyslipidaemia, metabolic dysfunction associated fatty liver disease, metabolic syndrome), even in the absence of iron deficiency anaemia or iron overload [1,2,3,4,5,6]. A relationship between serum iron, hepcidin, ferritin, and transferrin saturation with the risk of type 2 diabetes has been described [7,8,9,10,11]. Also, numerous studies have linked dietary iron intake to deranged glucose homeostasis, including changes in plasma concentrations of markers of glucose metabolism—fasting plasma glucose (FPG) and haemoglobin A1c (HbA1c) [7,8,9,10,11].

Deranged glucose metabolism is a frequent sequela of acute pancreatitis (AP)—the most common disease of the exocrine pancreas [12,13,14,15]. Altered levels of circulating markers of iron metabolism—decreased ferritin and increased hepcidin—have been reported in individuals with chronic hyperglycaemia after an attack of AP [16]. While hyperglycaemia in AP was historically deemed to be a brief and transient consequence of metabolic stress associated with acute illness, a comprehensive meta-analysis of clinical cross-sectional and case-control studies has shown that new-onset prediabetes or diabetes mellitus develops in 40% of individuals after their first attack of AP [12]. Also, population-based studies have shown that the risk of new-onset diabetes increases more than two-fold after AP in comparison with the general population [17]. Further, in the first prospective longitudinal cohort study involving individuals after an attack of AP (without pre-existing diabetes), derangements in glucose metabolism were shown to occur progressively after AP [18]. Specifically, the study reported that HbA1c levels increased from baseline by 24% at 12 months and 37% at 24 months of follow-up [18]. However, investigations into the pathogenesis of changes in glucose metabolism following AP have only begun to gain momentum [19]. It is conceivable that iron metabolism and glucose homeostasis have a bidirectional relationship where the altered intake of iron is linked with deranged markers of glucose metabolism [20]. Further, the gut-iron regulatory pathway may be more accentuated in the post-pancreatitis setting, as evidenced by reports of diminished iron uptake in individuals with pancreatitis [21,22,23]. Given the above arguments, we hypothesised that habitual dietary iron intake plays a role in the dysregulation of blood glucose homeostasis after an attack of AP.

The aim of the present study was to investigate the association between dietary iron and its components (i.e., haem and non-haem) and markers of glucose metabolism (i.e., FPG and HbA1c) in individuals after an attack of AP.

## 2. Materials and Methods

### 2.1. Study Design

The study was a cross-sectional investigation of patients after an attack of AP as part of the ANDROMEDA project. Ethics approval was issued by the Health and Disability Ethics Committee (13/STH/182). Individuals were eligible to participate if they were ≥ 18 years of age, lived in Auckland at the time of the study, provided informed consent, and had a primary diagnosis of AP established prospectively. Diagnosis of AP was established at the time of hospitalisation to Auckland City Hospital in line with the most up-to-date international guidelines that stipulate the fulfilment of at least two of the three criteria: (1) levels of serum lipase, amylase, and/or pancreatic amylase at least three times the upper limit of the normal range, (2) pain indicative of AP, (3) transabdominal ultrasonography or computed tomography findings suggestive of AP [24]. Individuals were excluded from the study if they had chronic pancreatitis, intraoperative diagnosis of pancreatitis, post-endoscopic retrograde cholangiopancreatography pancreatitis, pregnancy at the time of AP or afterwards, malignancy, history of steroid use, celiac disease, or cystic fibrosis.

### 2.2. Ascertainment of Dietary Intake

Intake of individuals’ habitual diet over the year prior to recruitment was assessed using the EPIC-Norfolk food frequency questionnaire (FFQ) [25]. Extensive validation of the FFQ enabled the collection of frequency and portion sizes of 130 food items. Daily amounts of regularly consumed nutrients and foods were determined using the FETA software to calculate the intake of total, haem, and non-haem iron (mg), as well as energy (kcal) and alcohol (g) [25]. FFQs were excluded if more than 10 food items were left unanswered as this level of missing data would lead to a considerable underestimate of intake [25]. In addition, if the total energy intake estimates from the FFQ data (as a ratio of the estimated basal metabolic rate determined by the Harris-Benedict equation) were more than two standard deviations (SD) outside the mean ratio, (i.e., less than −0.50 or more than 2.74), FFQ data were excluded [25]. The ascertainment of dietary intake in this study was restricted to the assessment of individual nutrients from food intake only; hence, the intake of nutrients from dietary supplements was not considered.

### 2.3. Markers of Glucose Metabolism

All participants were required to fast 8 h prior to blood collection. Venous blood samples were collected in EDTA tubes, then centrifuged at 4000× *g* for 7.5 min. Blood tests for HbA1c (mmol/mol) were conducted immediately after blood collection on fresh and never frozen blood using the borate affinity chromatography assay (Trinity Biotech, Wicklow, Ireland), which is standardised to the Diabetes Control and Complications Trial reference assay and certified by the U.S. National Glycohaemoglobin Standardisation Program, at LabPlus (Auckland City Hospital). FPG (mmol/L) tests were conducted in the same laboratory using an enzymatic colourimetric assay (F.Hoffmann-La Roche Ltd., Basel, Switzerland).

### 2.4. Definitions of Covariates

Data regarding age and sex were collected during a standardised face-to-face health examination by the research team. The use of anti-diabetic medications was derived from participants’ health records. Anthropometric data (height and weight) were measured according to standard protocols using a digitalised medical scale with a stadiometer (Health o meter^®^ Professional 2013, Pelstar LLC, McCook, IL, USA). Body mass index (BMI) (kg/m^2^) was calculated in as weight in kilograms divided by the square of the height in metres, and categorised as underweight if BMI < 18.5 kg/m^2^, normal weight with a BMI between 18.5 to <25 kg/m^2^, overweight with a BMI between 25.0 to <30 kg/m^2^, and obese with a BMI of ≥30 kg/m^2^. Tobacco smoking status was recorded as ‘yes’ or ‘no’ based on a previously validated standardised questionnaire asking participants whether they smoked cigarettes or tobacco-related products daily [26]. Alcohol consumption (g) and energy intake (kcal) were assessed using the EPIC Norfolk FFQ and determined using the FETA software [25]. Triglycerides were measured at LabPlus. Recurrence of AP was defined as a new hospitalisation for AP since the first admission, with a minimum 30-day period of resolution between the episodes. Aetiology of AP was characterised as biliary or non-biliary (including alcohol-related and other).

### 2.5. Statistical Analyses

All statistical analyses were performed using SAS 9.4. (SAS Institute Inc., Cary, NC, USA). Data on characteristics of study participants were presented as mean and SD, or frequency and percentage (%). All *p* values were two-tailed, and if less than 0.05, were deemed statistically significant. The *p* values reported were corrected for multiple comparisons using the Benjamini-Hochberg procedure [27]. In the analyses, data were presented as β coefficients and 95% confidence intervals (CI).

Total dietary iron, haem, and non-haem iron were treated as exposures. Multivariable linear regression analyses were conducted to investigate the associations between HbA1c and FPG and dietary iron (total, haem, and non-haem iron) in the overall cohort. Normality assessment using the Shapiro-Wilk test showed the skewed distribution of total iron intake, haem iron, non-haem iron, HbA1c, FPG, energy, and triglycerides; hence, these variables were logarithmically transformed. A total of 6 models were built in a step-wise manner for all the linear regression analyses. Model 1 was an unadjusted model; Model 2 was adjusted for energy intake (kcal); Model 3 was adjusted for the variables included in Model 2 and for patient-related characteristics (age, sex, BMI category); Model 4 was adjusted for the variables included in Model 3 and for lifestyle characteristics (alcohol consumption and tobacco smoking); Model 5 was adjusted for the variables included in Model 4 and for triglycerides; and Model 6 was adjusted for the variables included in Model 5 and for the use of anti-diabetic medications.

Linear regression models (analogous to those applied to the overall cohort) were built to conduct sub-group analyses with a view to investigating the associations between FPG/HbA1c and dietary iron in the sub-groups stratified by aetiology (biliary versus non-biliary AP) and recurrence of AP (first episode of acute pancreatitis (FAP) versus recurrent acute pancreatitis (RAP)).

## 3. Results

### 3.1. Study Cohort

A total of 109 individuals were included in the study in a mean ± SD of 26.0 ± 19.2 months after an attack of AP. Descriptive characteristics of participants are presented in Table 1. The mean ± SD of dietary iron intake in the overall cohort was as follows: total iron = 10.16 ± 4.14 mg/day, haem iron = 0.87 ± 0.45 mg/day, and non-haem iron = 9.29 ± 3.94 mg/day. Non-haem iron constituted 91.4 % of total iron intake in the overall cohort. The level of FPG was 5.86 ± 1.64 mmol/L, whereas that of HbA1c was 40.03 ± 10.67 mmol/mol in the overall cohort.

### 3.2. Association between Fasting Plasma Glucose and Dietary Iron

**Figure 1:**
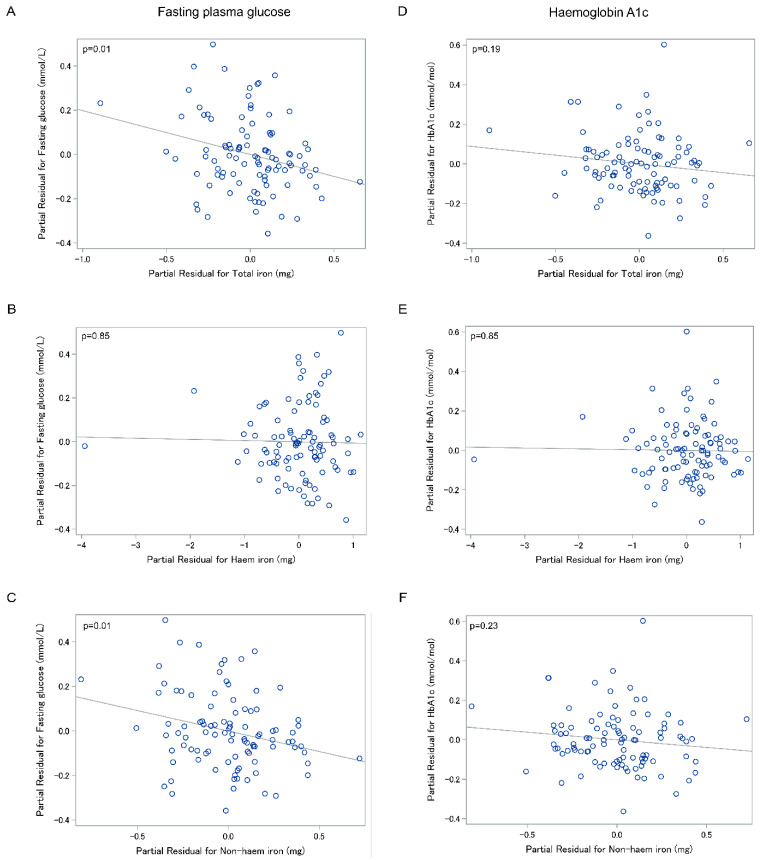
Associations between fasting plasma glucose and total iron intake (**A**), haem iron (**B**), and non-haem iron (**C**); haemoglobin A1c and total iron intake (**D**), haem iron (**E**), and non-haem iron (**F**) in the entire cohort. Footnote: All partial residual plots were adjusted for energy (kcal), age, sex, body mass index category, tobacco smoking, alcohol consumption, triglycerides, and use of anti-diabetic medications.

In the entire cohort, total iron intake was inversely significantly associated with FPG in all the adjusted models (Figure 1), but not in the unadjusted model (Table 2). In the most adjusted model (model 6), the β coefficient (95% CI) was −0.19 (−0.35, −0.05); *p* = 0.02. There was no significant association between haem iron and FPG in both unadjusted and adjusted models. Non-haem iron was inversely significantly associated with FPG in all the adjusted models, but not in the unadjusted models. In the most adjusted model (model 6), the β coefficient (95% CI) was −0.19 (−0.33, −0.04); *p* = 0.03.

In participants with biliary AP, total iron intake was inversely significantly associated with FPG in both the unadjusted and adjusted models. In the most adjusted model (model 6), the β coefficient (95% CI) was −0.39 (−0.63, −0.16); *p* < 0.01. There was no significant association between haem iron and FPG in all models. Non-haem iron was inversely significantly associated with FPG in both unadjusted and adjusted models. In the most adjusted model, the β coefficient (95% CI) was −0.39 (−0.61, −0.18); *p* < 0.01. In participants with non-biliary AP, there was no significant association between FPG and dietary iron in all models (Table 3).

In FAP participants, total iron intake was inversely significantly associated with FPG. In the most adjusted model (model 6), the β coefficient (95% CI) was −0.23 (−0.42, −0.05); *p* = 0.02. There was no significant association between FPG and haem iron in both the adjusted and unadjusted models. Non-haem iron was inversely significantly associated with FPG in all the adjusted models, but not in the unadjusted model. In the most adjusted model (model 6), the β coefficient (95% CI) was −0.23 (−0.40, −0.05); *p* = 0.02. In RAP participants, there was no significant association between FPG and dietary iron in all models (Table 4).

### 3.3. Association between Haemoglobin A1c and Dietary Iron

There was no significant association between HbA1c and dietary iron in the overall cohort (Table 2, Figure 1) as well as the sub-groups stratified by aetiology (Table 3) and AP recurrence (Table 4), in both the unadjusted and adjusted models. 

## 4. Discussion

The present study was the first to investigate the association between habitual dietary iron intake and markers of glucose metabolism (FPG and HbA1c) in individuals with history of pancreatitis. It found a significant inverse association between total iron, non-haem iron intake and FPG (but not HbA1c) in the overall cohort. At the same time, dietary haem iron intake was not associated with either FPG or HbA1c. Further, individuals after biliary AP (but not non-biliary) and those with FAP (but not RAP) had a significant inverse association between total iron, non-haem iron intake and FPG (but not HbA1c).

There has been no lack of studies on dietary iron intake and markers of glucose metabolism [2,3,28,29,30,31]. However, they have produced differing results and currently there is no consensus on whether or not habitual dietary iron intake affects blood glucose metabolism. Part of the reason is that prior studies exploring the relationship between dietary iron intake and blood markers of glucose metabolism varied greatly in terms of the methods used to measure blood markers of glucose metabolism and to ascertain dietary iron intake. In the present study, both FPG and HbA1c were quantified, enabling more comprehensive glycaemic profiling. While HbA1c quantifies blood glucose levels over the previous 8–12 weeks (hence, mitigating the day-to-day intra-individual variations in plasma glucose), FPG is specific to glucose levels in plasma after fasting and is, therefore, unaltered by erythrocyte abnormalities (such as hemoglobinopathies), which may lower HbA1c independently of glycaemia [32]. Further, laboratory analyses of both HbA1c and FPG were carried out in a tertiary hospital laboratory using the methods recommended by the American Diabetes Association [33]. Also, all the analyses were done on fresh and never frozen blood in line with the guidelines [19]. A statistically significant relationship between blood markers of glucose metabolism and dietary iron would be missed if only HbA1c was used in the present study. Dietary intake data were collected using a widely used FFQ [25], which gives a better representation of habitual dietary intake (over 12 months) of nutrients and foods compared with shorter-term measurements (such as 24 h recalls and 3-day food records) [34]. Another important aspect to consider in studying the relationship between dietary iron intake and glucose metabolism is the study population. While previous studies investigated the relationship in individuals with anaemia, metabolic syndrome, and type 2 diabetes, the present study was the first to investigate this relationship in a unique and relatively homogenous cohort of individuals after an attack of AP. This study population is important because diseases of the exocrine pancreas affect gut function (including but not limited to absorption of nutrients) [21,22,23] and often lead to metabolic sequelae (including but not limited to post-pancreatitis diabetes mellitus, intra-pancreatic fat deposition, gout, liver disease) [12,35,36,37,38].

To elucidate the relationship between dietary iron and glucose metabolism in our study population, we analysed not merely total iron, but also the two forms of dietary iron—haem (organic, Fe^+^) and non-haem (inorganic, Fe^3+^)—separately. This was because of their well-acknowledged differences in chemical structure, food sources, and absorptive properties in the gut [39]. Haem iron is typically found in foods of animal origin (e.g., red meat, fish, chicken) in the form of haemoglobin or myoglobin, whereas non-haem iron is found in foods of plant origin. While around 20% of haem iron is absorbed from a typical Western diet, only around 2% of non-haem iron is absorbed (the two forms are absorbed non-competitively) [39]. This is because haem is taken up as an intact metalloporphyrin by intestinal cells through endocytosis, whereas ferric iron needs to be transformed by duodenal reductases and transported by divalent metal transporter 1 (a protein that also transports other metallic ions, such as zinc, copper, and cobalt). In the context of our study in individuals after an attack of AP, it is also pertinent to note that haem is maintained soluble and readily available for absorption by globin degradation products produced by pancreatic enzymes [40,41]. Dietary non-haem iron made up 91.4% of total iron intake in the present study, which is well in line with a typical Western diet [42]. We found that dietary haem iron intake was not associated with FPG in our study population, corroborating findings from large population-based studies in other disease settings [30,43]. By contrast, there was a significant inverse association between non-haem iron intake and FPG. This finding was novel and was contrary to previous studies reporting either a significant direct association [1,2] or no significant association [3,28]. We speculate that our findings may be attributed not to the effect of systemic iron homeostasis, but to the effect of non-absorbable non-haem iron in the gut (expanding into the gut-brain axis and gut-pancreas axis). This is because, under physiological conditions, only around 5–10% of daily habitual intake of iron (i.e., around 1 mg/day) is absorbed in the duodenum and the first part of the jejunum. Of note, normal body loss of iron predominantly occurs when intestinal cells (born in the crypts of Lieberkuhn) migrate to the tips of the villi and get sloughed from the gut lining at the end of their 48–72 h lifespan, and this process is quantitatively not dissimilar to iron absorption [44]. Moreover, our earlier study specifically in individuals after an attack of AP (as part of the DORADO project) showed that circulating levels of hepcidin—the master regulator of intestinal absorption of iron—are significantly increased in individuals with high FPG [16]. Notably, lipopolysaccharide-binding protein (which adheres to lipopolysaccharide of gut-derived gram-negative bacteria and enhances the immunostimulatory capacity of lipopolysaccharide towards monocytes and granulocytes) was significantly directly associated with hepcidin and explained 7.7% of its variance [45]. In addition, earlier studies by other research groups that investigated radioactivity in faeces after the ingestion of a test meal containing radioactive iron showed a significantly reduced iron absorption in pancreatitis [21,22]. Non-absorbable non-haem iron in the gut could be involved in the regulation of blood glucose control via several putative intermediaries, including but not limited to gut microbiota, short-chain fatty acids, and/or bile acids [46].

The possible role of bile acids in the observed association is supported by the finding of a significant inverse association between total iron and non-haem iron intake and FPG only in individuals after biliary, but not non-biliary, AP. Given that 70% (34/48) of individuals after biliary AP underwent cholecystectomy following their first episode of AP—known to prevent AP recurrence [13,47], individuals who underwent cholecystectomy contributed markedly to the FAP subgroup, in which a significant inverse association between total dietary iron and non-haem iron intake and FPG was maintained. As the present study was the first to make this observation, we can only speculate about the underlying mechanisms. One possibility is that cholecystectomised individuals may have an altered glucose metabolism owing to bile acid dysregulation. Studies showed that bile acids play a key role in the release of fibroblast growth factor−19 (FGF19)—a hormone that suppresses liver gluconeogenesis and stimulates glucose uptake in adipocytes [48]. Further, there is evidence that, in addition to the ileum, the cholangiocytes also secrete FGF19 [49]. Cholecystectomy has been shown to lead to decreased levels of FGF19 [50] that result in a disruption in the transintestinal flow of bile acids triggering abnormal metabolic signalling in the liver, adipose tissue, muscle, and gut [48]. There is evidence showing that resection of the gallbladder also increases the enterohepatic recirculation rates of bile acids, increasing the exposure of the bile acid pool to intestinal bacteria, which alters gut microbiota causing gut dysbiosis [51]. Given that dysbiosis is posited to alter glucose metabolism [52] and, at the same time, is affected by dietary intake of iron [53], it appears that dietary iron intake may be an important factor in the genesis of abnormalities of glucose metabolism in cholecystectomised individuals after an attack of AP. However, further research is warranted to elucidate the exact role of iron in the gallbladder-gut-glucose metabolism link in individuals after an attack of AP.

The results reported herein should be considered in light of several limitations. First, dietary intake was self-reported and, hence, we cannot rule out the possibility of a measurement error. However, we used a validated FFQ that gives a better estimation of habitual dietary intake of iron when compared with previously used short-term assessment methods, such as 24 h dietary records [25,34]. Second, several possible confounders were not accounted for. For example, medications (e.g., proton pump inhibitors, antacids, and tetracycline) are known to reduce iron absorption. Also, several dietary factors (e.g., meat, vitamin C, citric acid, dairy, calcium, phytates, oxalates, tannins) may affect the bioavailability of non-haem iron. Nonetheless, we adjusted for numerous confounders of the studied association, including age, sex, BMI category, lifestyle factors, such as tobacco smoking and alcohol use, and the use of anti-diabetic medications, providing a robust model. Third, malabsorption of iron might have affected the studied association. However, it typically occurs in small bowel diseases (e.g., celiac disease, enteritis) or following gastrointestinal surgery—neither of which was present in any of the individuals in our study cohort. Fourth, our findings require validation. Future studies may consider external validation of our findings. Also, as is the case with all cross-sectional studies, one cannot infer causality. Therefore, there is a need for prospective longitudinal cohort studies to provide deeper insights into the cause and effect relationship between the studied variables. However, this was the first study highlighting the association between habitual dietary iron intake and glucose metabolism in a unique cohort of individuals after an attack of AP. Last, iron overload and iron deficiency anaemia might have affected the studied associations. Also, circulating markers of iron metabolism were not available in the present study. However, our earlier study in individuals after an attack of AP (as part of the DORADO project) showed that ferritin levels were significantly decreased and soluble transferrin receptor levels were unchanged [16], indicating that iron overload and iron deficiency anaemia (respectively) are not prevalent in the studied population.

## 5. Conclusions

Habitual non-haem (and total) iron intake was significantly associated with FPG in individuals following AP. This was pronounced in those with biliary aetiology of AP. The above findings provide a platform for future studies to investigate the mechanisms underlying the complex involvement of dietary iron into the pathogenesis of deranged glucose metabolism after an attack of pancreatitis.

## Figures and Tables

**Table 1:** Characteristics of the study participants.

Characteristic	Mean (SD) or *n* (%)
Age (years)	56.22 (14.82)
Men	73 (70)
BMI category	
<18.5 kg/m^2^	1 (1)
18.5–25 kg/m^2^	32 (29)
25.1–29.9 kg/m^2^	38 (35)
≥30 kg/m^2^	38 (35)
Tobacco smoking	
Yes	45 (43)
No	59 (57)
Alcohol consumption (g/day)	11.41 (18.21)
Haemoglobin A1c (mmol/mol)	40.03 (10.67)
Fasting plasma glucose (mmol/L)	5.86 (1.64)
Triglycerides (mmol/L)	2.16 (3.06)
Aetiology	
Biliary	58 (58)
Non-biliary	42 (42)
Recurrence	
Yes	31 (30)
No	71 (70)
Energy (kcal/day)	1734.94 (674.43)
Total iron (mg/day)	10.16 (4.14)
Haem iron (mg/day)	0.87 (0.45)
Non-haem iron (mg/day)	9.29 (3.94)

Abbreviations: SD, standard deviation; BMI body mass index.

**Table 2:** Association between (A) fasting plasma glucose, (B) haemoglobin A1c and dietary iron in the overall cohort.

		(A) Fasting Plasma Glucose			(B) Haemoglobin A1c		
Dietary Iron							
	Model	*n*	β Coefficient (95% CI)	*p*	*n*	β Coefficient (95% CI)	*p*
Total iron (mg)	1	109	−0.05 (−0.15, 0.05)	0.30	108	−0.03 (−0.12, 0.07)	0.60
	2	109	−0.27 (−0.43, −0.10)	**<0.01**	108	−0.19 (−0.35, 0.03)	0.09
	3	109	−0.25 (−0.41, −0.09)	**<0.01**	108	−0.17 (−0.33, −0.02)	0.09
	4	104	−0.24 (−0.42, −0.07)	**0.01**	103	−0.16 (−0.32, 0.01)	0.12
	5	100	−0.21 (−0.38, −0.05)	**0.01**	99	−0.13(−0.29, 0.03)	0.17
	6	100	−0.19 (−0.35, −0.05)	**0.01**	99	−0.09 (−0.22, 0.04)	0.23
Haem iron (mg)	1	109	<−0.01 (−0.06, 0.06)	0.18	108	<−0.01 (−0.06, 0.05)	0.99
	2	109	−0.01 (−0.08, −0.05)	0.08	108	−0.02 (−0.08, 0.05)	0.99
	3	109	−0.02 (−0.08, −0.04)	0.08	108	−0.02 (−0.08, 0.04)	0.99
	4	104	−0.01 (−0.08, 0.06)	0.10	103	−0.01 (−0.07, 0.05)	0.99
	5	100	−0.01 (−0.07, 0.05)	0.18	99	−0.01 (−0.07, 0.05)	0.99
	6	100	−0.01 (−0.06, 0.05)	0.34	99	−0.01 (−0.06, 0.04)	0.99
Non-haem iron (mg)	1	109	−0.04 (−0.14, 0.05)	0.35	108	−0.02 (−0.11, 0.07)	0.71
	2	109	−0.24 (−0.40, −0.08)	**0.02**	108	−0.16 (−0.31, 0.01)	0.18
	3	109	−0.22 (−0.37, −0.06)	**0.02**	108	−0.14 (−0.29, 0.01)	0.18
	4	104	−0.21 (−0.38, −0.05)	**0.03**	103	−0.13 (−0.29, 0.03)	0.21
	5	100	−0.19 (−0.35, −0.03)	**0.03**	99	−0.11 (−0.26, 0.05)	0.24
	6	100	−0.19 (−0.33, −0.04)	**0.03**	99	−0.08 (−0.20, 0.04)	0.24

Abbreviation: CI, Confidence Interval. Footnotes: Model 1: Unadjusted; Model 2: Adjusted for energy (kcal); Model 3: Adjusted for energy (kcal), age, sex, body mass index category; Model 4: Adjusted energy (kcal), age, sex, body mass index category, tobacco smoking, alcohol consumption; Model 5: Adjusted for energy (kcal), age, sex, body mass index category, tobacco smoking, alcohol consumption, triglycerides; Model 6: Adjusted for energy (kcal), age, sex, body mass index category, tobacco smoking, alcohol consumption, triglycerides, use of anti-diabetic medications. *p* values were corrected for multiple comparisons. *p* values < 0.05 are shown in bold.

**Table 3:** Association between (A) fasting plasma glucose, (B) haemoglobin A1c and dietary iron in the sub-groups stratified by aetiology of acute pancreatitis.

		(A) Fasting Plasma Glucose						(B) Haemoglobin A1c					
Dietary Iron		Biliary			Non-Biliary				Biliary			Non-Biliary	
	Model	*n*	β Coefficient (95% CI)	*p*	*n*	β Coefficient (95% CI)	*p*	*n*	β Coefficient (95% CI)	*p*	*n*	β Coefficient (95% CI)	*p*
Total iron (mg)	1	42	−0.16 (0.28, −0.05)	**0.01**	58	0.04 (−0.12, 0.20)	0.68	42	−0.10 (−0.20, −0.01)	0.06	58	0.04 (−0.12, 0.20)	0.65
	2	42	−0.35 (−0.54, −0.17)	**<0.01**	58	−0.16 (−0.44, 0.12)	0.68	42	−0.17 (−0.33, 0.01)	0.06	58	−0.17 (−0.46, 0.12)	0.65
	3	42	−0.35 (−0.54, −0.16)	**<0.01**	58	−0.11 (−0.39, 0.17)	0.68	42	−0.18 (−0.34, −0.01)	0.06	58	−0.11 (−0.39, 0.17)	0.65
	4	41	−0.35 (−0.53, −0.16)	**<0.01**	57	−0.08 (−0.38, 0.22)	0.68	41	−0.17 (−0.34, 0.01)	0.06	57	−0.08 (−0.38, 0.22)	0.65
	5	39	−0.38 (−0.60, −0.15)	**<0.01**	55	−0.10 (−0.36, 0.17)	0.68	39	−0.23 (−0.44, −0.03)	0.06	55	−0.11 (−0.38, 0.16)	0.65
	6	39	−0.39 (−0.63, −0.16)	**<0.01**	55	−0.05 (−0.27, 0.18)	0.68	39	−0.17 (−0.36, 0.01)	0.06	55	−0.05 (−0.26, 0.17)	0.65
Haem iron (mg)	1	42	−0.04 (−0.11, 0.04)	0.60	58	0.01 (−0.08, 0.10)	0.91	42	−0.04 (−0.10, 0.02)	0.28	58	0.03 (−0.07,0.12)	0.72
	2	42	−0.03 (−0.12, 0.06)	0.65	58	−0.02 (−0.11, 0.08)	0.91	42	−0.03 (−0.10, 0.04)	0.44	58	<0.01 (−0.10, 0.10)	0.99
	3	42	−0.06 (−0.15, 0.03)	0.60	58	0.02 (−0.08, 0.13)	0.91	42	−0.07 (−0.14, 0.01)	0.13	58	0.05 (−0.05, 0.15)	0.72
	4	41	−0.05 (−0.15, 0.04)	0.60	57	0.03 (−0.07, 0.14)	0.91	41	−0.07 (−0.14, 0.01)	0.13	57	0.06 (−0.05, 0.16)	0.72
	5	39	−0.03 (−0.13, 0.07)	0.65	55	0.02 (−0.07, 0.11)	0.91	39	−0.08 (−0.16, 0.01)	0.13	55	0.04 (−0.05, 0.14)	0.72
	6	39	0.01 (−0.10, 0.12)	0.92	55	0.01 (−0.07, 0.08)	0.91	39	−0.03 (−0.11, 0.05)	0.44	55	0.02 (−0.06, 0.09)	0.72
Non-haem iron (mg)	1	42	−0.17 (−0.28, −0.05)	**<0.01**	58	0.06 (−0.09, 0.21)	0.80	42	−0.11 (−0.20, −0.01)	0.10	58	0.05 (−0.10, 0.20)	0.64
	2	42	−0.36 (−0.54, −0.18)	**<0.01**	58	−0.09 (−0.36, 0.17)	0.80	42	−0.16 (−0.32, 0.01)	0.10	58	−0.12 (−0.39, 0.15)	0.64
	3	42	−0.34 (−0.53, −0.16)	**<0.01**	58	−0.07 (−0.33, 0.19)	0.80	42	−0.16 (0.32, 0.01)	0.10	58	−0.09 (−0.35, 0.17)	0.64
	**4**	41	−0.34 (−0.53, −0.16)	**<0.01**	57	−0.04 (−0.32, 0.23)	0.80	41	−0.14 (−0.31, 0.03)	0.10	57	−0.07 (−0.34, 0.21)	0.64
	**5**	39	−0.36 (−0.58, −0.15)	**<0.01**	55	−0.07 (−0.32, 0.17)	0.80	39	−0.19 (−0.39, 0.02)	0.10	55	−0.11 (−0.36, 0.14)	0.64
	**6**	39	−0.39 (−0.61, −0.18)	**<0.01**	55	−0.03 (−0.23, 0.18)	0.80	39	−0.16 (−0.33, 0.02)	0.10	55	−0.05 (−0.25, 0.15)	0.64

Abbreviation: CI, Confidence Interval. Footnotes: Model 1: Unadjusted; Model 2: Adjusted for energy (kcal); Model 3: Adjusted for energy (kcal), age, sex, body mass index category; Model 4: Adjusted energy (kcal), age, sex, body mass index category, tobacco smoking, alcohol consumption; Model 5: Adjusted for energy (kcal), age, sex, body mass index category, tobacco smoking, alcohol consumption, triglycerides; Model 6: Adjusted for energy (kcal), age, sex, body mass index category, tobacco smoking, alcohol consumption, triglycerides, use of anti-diabetic medications. *p* values were corrected for multiple comparisons. *p* values < 0.05 are shown in bold.

**Table 4:** Association between (A) fasting plasma glucose, (B) haemoglobin A1c and dietary iron in sub-groups stratified by the recurrence of acute pancreatitis.

		(A) Fasting Plasma Glucose						(B) Haemoglobin A1c					
Dietary Iron			FAP			RAP			FAP			RAP	
	Model	*n*	β Coefficient (95% CI)	*p*-Value	*n*	β Coefficient (95% CI)	*p*-Value	*n*	β Coefficient (95% CI)	*p*-Value	*n*	β Coefficient (95% CI)	*p*-Value
Total iron (mg)	1	71	−0.03 (−0.13, 0.07)	0.58	31	−0.09 (−0.33, 0.16)	0.48	72	0.02 (−0.08, 0.11)	0.71	30	−0.10 (−0.35, 0.14)	0.40
	2	71	−0.26 (−0.44, −0.07)	**0.02**	31	−0.30 (−0.69, 0.08)	0.26	72	−0.09 (−0.26, 0.09)	0.71	30	−0.30 (−0.68, 0.08)	0.20
	3	71	−0.27 (−0.45, −0.08)	**0.02**	31	−0.33 (−0.71, 0.05)	0.26	72	−0.09 (−0.27, 0.08)	0.71	30	−0.33 (−0.70, 0.05)	0.20
	4	68	−0.26 (−0.46, −0.07)	**0.02**	31	−0.29 (−0.69, 0.11)	0.26	69	−0.08 (−0.27, 0.10)	0.71	30	−0.29 (−0.68, 0.10)	0.20
	5	65	−0.25 (−0.46, −0.05)	**0.02**	30	−0.25 (−0.62, 0.12)	0.26	66	−0.06 (−0.25, 0.14)	0.71	29	−0.28 (−0.64, 0.09)	0.20
	6	65	−0.23 (−0.42, −0.05)	**0.02**	30	−0.13 (−0.38, 0.13)	0.39	66	−0.04 (−0.19, 0.12)	0.71	29	−0.16 (−0.45, 0.13)	0.30
Haem iron (mg)	1	71	−0.01 (−0.07, 0.04)	0.98	31	0.03 (−0.14, 0.20)	0.84	72	<0.01 (−0.05, 0.06)	0.97	30	0.01 (−0.16, 0.17)	0.96
	2	71	−0.03 (−0.09, 0.03)	0.98	31	0.02 (−0.20, 0.24)	0.84	72	<−0.01 (−0.07,0.05)	0.97	30	−0.01 (−0.23, 0.20)	0.96
	3	71	−0.02 (−0.08, 0.04)	0.98	31	−0.06 (−0.29, 0.18)	0.84	72	−0.01 (−0.06, 0.06)	0.97	30	−0.10 (−0.33, 0.14)	0.79
	4	68	−0.01 (−0.07, 0.06)	0.98	31	−0.10 (−0.36, 0.15)	0.84	69	<0.01 (−0.06, 0.06)	0.97	30	−0.09 (−0.34, 0.15)	0.79
	5	65	−0.01 (−0.07, 0.06)	0.98	30	−0.12 (−0.35, 0.11)	0.84	66	0.01 (−0.05, 0.07)	0.97	29	−0.12 (−0.36, 0.11)	0.79
	6	65	−0.01 (−0.06, 0.06)	0.98	30	−0.19 (−0.67, 0.29)	0.84	66	0.01 (−0.04, 0.05)	0.97	29	−0.06 (−0.24, 0.13)	0.79
Non-haem iron (mg)	1	71	−0.03 (−0.12, 0.07)	0.59	31	−0.07 (−0.32, 0.18)	0.57	72	0.02 (−0.07,0.11)	0.70	30	−0.09 (−0.34, 0.16)	0.47
	2	71	−0.23 (−0.40, −0.06)	**0.02**	31	−0.26 (−0.65, 0.13)	0.41	72	−0.08 (−0.24, 0.09)	0.67	30	−0.26 (−0.64, 0.12)	0.30
	3	71	−0.25 (−0.42, −0.07)	**0.02**	31	−0.28 (−0.66, 0.10)	0.41	72	−0.11 (−0.26, 0.07)	0.67	30	−0.28 (−0.66, 0.09)	0.30
	4	68	−0.25 (−0.43, −0.07)	**0.02**	31	−0.23 (−0.64, 0.17)	0.41	69	−0.08 (−0.26, 0.09)	0.67	30	−0.25 (−0.65, 0.14)	0.30
	5	65	−0.24 (−0.43, −0.05)	**0.02**	30	−0.20 (−0.58, 0.17)	0.41	66	−0.06 (−0.24, 0.12)	0.67	29	−0.24 (−0.62, 0.13)	0.30
	6	65	−0.23 (−0.40, −0.05)	**0.02**	30	−0.12 (−0.43, 0.19)	0.50	66	−0.04 (−0.19, 0.10)	0.67	29	−0.16 (−0.45, 0.12)	0.30

Abbreviations: CI, Confidence Interval; FAP, First Episode of Acute Pancreatitis; RAP, Recurrent Episodes of Acute Pancreatitis. Footnotes: Model 1: Unadjusted; Model 2: Adjusted for energy (kcal); Model 3: Adjusted for energy (kcal), age, sex, body mass index category; Model 4: Adjusted energy (kcal), age, sex, body mass index category, tobacco smoking, alcohol consumption; Model 5: Adjusted for energy (kcal), age, sex, body mass index category, tobacco smoking, alcohol consumption, triglycerides; Model 6: Adjusted for energy (kcal), age, sex, body mass index category, tobacco smoking, alcohol consumption, triglycerides, use of anti-diabetic medications. *p* values were corrected for multiple comparisons. *p* values < 0.05 are shown in bold.
